# The association between temporomandibular disorders and kinematics of the sacroiliac joint: a 3D motion analysis study

**DOI:** 10.1186/s12891-025-08717-6

**Published:** 2025-06-02

**Authors:** Leila Rahnama, Ahmad Bahramian Parchekouhi, Noureddin Karimi, Ali barzegar

**Affiliations:** 1 School of Kinesiology, Rongxiang Xu College of Health and Human Services, California State University, Los Angeles, Los Angeles, CA USA; 2 Department of Physiotherapy, University of Social Welfare and Rehabilitation Sciences, Tehran, Iran; 3 Shahid Sadoughi University of Medical Sciences, Yazd, Iran

**Keywords:** Temporomandibular disorders, Sacroiliac joint – kinematics, Motion analysis system

## Abstract

**Background:**

Temporomandibular disorders (TMDs) have been the focus of many studies, but the relationship between TMDs and other body segments, such as the pelvis, remains controversial. This study aimed to investigate the relationship between TMD and kinematics of the sacroiliac joint.

**Method:**

Twenty participants with chronic TMD and 20 healthy individuals were included in this study. The temporomandibular joint range of motion, pain intensity, and TMD severity were documented. The three-dimensional kinematics of the sacroiliac joint were recorded during a trunk flexion task using a motion analysis system and MATLAB software.

**Results:**

The severity of TMD had a significant association with pain intensity in the patient group. At the starting position (before forward flexion of the trunk), we found significant differences in the linear and angular positions of the sacrum relative to the ilium in the sagittal plane and around the frontal axis on both sides between groups.

**Conclusion:**

Our results indicated significant differences in some parameters between the healthy and TMD groups, emphasizing altered sacroiliac joint kinematics associated with TMD. This study contributes to the ongoing discussion about the relationship between TMD and other areas of the body and highlights the importance of further research in this area.

**Trial registration:**

Clinical Trial number is not applicable.

## Background

Temporomandibular disorders (TMDs) is a comprehensive term referring to any symptom, such as clicking, limited range of motion or pain in the temporomandibular joint (TMJ), masticatory muscles or related structures [1]. These symptoms are significantly associated with stiffness in muscles during mastication, teeth grinding, other abnormal oral habits and psychosocial factors such as worry, stress, anger, despair and depression [2]. TMDs are classified along two axes: Axis I includes physical diagnoses, and Axis II addresses psychological status [3]. Our focus in this study is on axis I, where TMDs are further categorized into muscle disorders, disc displacements, and arthralgia [4]. Anterior disc displacement with reduction (ADDR) is the most common type of disk displacement in which the disc is displaced anteriorly when the person’s mouth is closed and by opening the mouth, the disc returns to its normal position in the joint, which may be accompanied by discomfort and clicking, popping, or crepitus [5].

The TMJ is linked to the cervical spine through myofascial and ligamentous connections, creating a functional relationship. TMJ disorders often lead to compensatory head postures, increasing cervical lordosis and contributing to body posture [6, 7]. Beyond the TMJ and cervical spine, similar anatomical and biomechanical links exist throughout the body. For instance, studies have demonstrated a bidirectional relationship between pelvic imbalances and TMD, including sacroiliac joint dysfunction, influencing distant regions such as the TMJ [8].

One proposed mechanism explaining the connection between the temporomandibular joint (as part of the cranium) and the sacroiliac region involves the attachments of the dura mater. The dura mater is anchored to the foramen magnum of the occipital bone and the first and second cervical vertebrae, without adhering to any other vertebrae along the spinal column. It then extends to the anterior aspect of the second sacral vertebra, potentially transmitting movements from the occiput to the sacrum [9].

Furthermore, research has identified associations between TMD and forward head posture [10] changes in neck mobility and pressure pain threshold in the neck muscles [11–13] as well as postural alterations in the longitudinal plantar arch and overall body alignment [14–19]. Another study that simulated TMD using an artificial device showed that in the simulated state, hypomobility occurs in both the upper cervical and the sacroiliac joints [20]. Despite evidence of these associations, the specific relationship between TMD and pelvic girdle kinematics remains underexplored. Previous studies have predominantly focused on clinical tests [10, 11, 15, 18, 20–23] or static photographs [24], which may not fully capture dynamic interactions between the TMJ, cervical spine, and pelvic girdle.

Given the pelvic girdle’s role as the body’s core and a stabilizer of the spine, dysfunction in this region can lead to widespread musculoskeletal disorders [8, 25]. To address this gap, the present study aims to objectively evaluate pelvic girdle kinematics in patients with TMD using a motion analysis system and to investigate the relationship between the two regions during a dynamic. By investigating sacroiliac joint kinematics, this research seeks to deepen our understanding of the biomechanical interplay between TMD and the pelvic girdle.

## Methods

This study was conducted in accordance with the Declaration of Helsinki and was approved by the Research Ethics Committee of the University of Social Welfare and Rehabilitation Sciences, Tehran, Iran (Ethics Code: IR.USWR.REC.1397.073). All participants signed the written informed consent, approved by the institutional IRB.

In total, forty individuals participated in this cross-sectional study: Twenty patient with chronic TMD (recruited from outpatient clinics and hospitals in Tehran, with a diagnosis of anterior disc displacement with reduction (ADDR) for a minimum of six months, confirmed by the Diagnostic Criteria for Temporomandibular Disorders (DC/TMD) [3]), and 20 healthy subjects (matched by sex, age and BMI) recruited from the Tehran community through advertisement, including flyers. Patients were introduced to the study and recruited during their regular clinic visits. Fifty-five cases were screened, among which 40 met the inclusion criteria and were selected for the study.

Patients were diagnosed with ADDR if in the last 30 days, any TMJ (temporomandibular joint) noise(s) present with jaw movement or function, OR patient report of any noise present during the exam. In addition to one of the above criteria, examination must reveal one of the following: clicking, popping and/or snapping noise during both opening and closing movements, detected with palpation during at least one of three repetitions of jaw opening and closing. OR clicking, popping and/or snapping noise detected with palpation during at least one of three repetitions of opening or closing movement(s) AND clicking, popping and/or snapping noise detected with palpation during at least one of three repetitions of right or left lateral, or protrusive movement(s) [3].

Considering the case-control design of the study and the type I error of α = 0.05 and the power of the test of β = 0.8, as well as considering the mean difference for the linear displacement variable of the sacrum relative to the ilium in a similar study [26], sample size was estimated. This variable was chosen because it closely reflects the physiological movement of the SIJ. The mean difference was 2.5 mm with a standard deviation of 1.5 mm. According to the following formula for calculating the sample size, the number of samples for each group was 19.51, which was rounded up to 20 samples per group:

$$\:\text{n}=\frac{{({z}_{1-\frac{\alpha\:}{2}}+{z}_{1-\beta\:})}^{2}({{\sigma\:}_{1}}^{2}+{{\sigma\:}_{2}}^{2})}{({{\mu\:}_{1}-{\mu\:}_{2})}^{2}}\approx\:20$$

After receiving the full explanation, the subjects signed the written consent form if they agreed. The inclusion criteria were no history of surgery in the temporomandibular joint (TMJ), spine, pelvic or lower limb; no low back pain in the past 30 days; and a normal body mass index (18 to 25 kg/m²). On the other hand, individuals with a lower limb discrepancy of more than 1 cm or with flat foot/feet, hypermobility syndrome (confirmed by the Beighton Hypermobility Index) [27], or abnormal spinal curves were excluded from the study. Piriformis or hamstring muscle tightness can impact sacroiliac joint mobility. Therefore, to control this factor, we evaluated the flexibility of these two muscles using a binary classification (“yes” or “no”) based on the protocol established by FP Kendall [28]. A “yes” indicated muscle shortness, while a “no” signified normal flexibility.

**Fig. 1:**
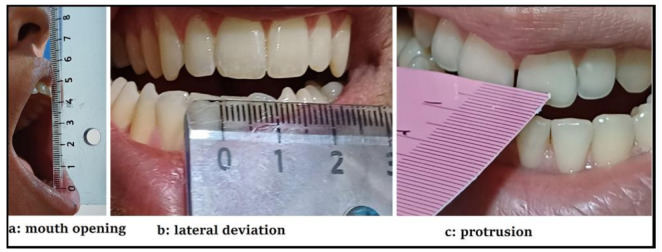
Range of Motion of the Temporomandibular Joint measured with a ruler

Outcome measures: Range of motion (ROM) of the TMJ, severity of TMD and pain in the patients’ group, and three-dimensional (3D) motions of the SIJ. The ROM of the TMJ, including the maximal mouth opening (Fig. 1-a), lateral deviation to the right and left (Fig. 1-b) and protrusion (Fig. 1-c), was measured with a ruler.

To assess the severity of TMD, we used the Fonseca Anamnestic Scale, which is a questionnaire that includes ten questions with three answers (no = 0, sometimes = 5, and yes = 10). This scale has been widely used to estimate the severity of TMD and has shown sufficient validity and reliability [29, 30]. The severity of TMJ pain was recorded using the visual analog scale (VAS). Highly reflective spherical markers (*n* = 10) (15 mm diameter) were used to define the anatomical landmarks of the pelvic girdle according to the study of Hungerford et al. [26].

**Fig. 2:**
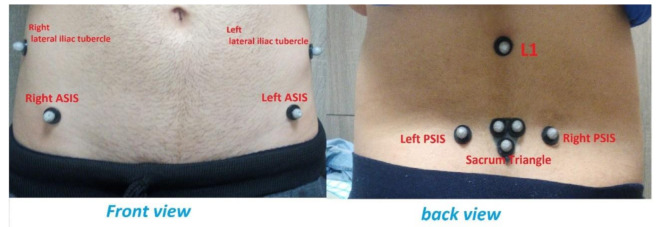
Marker set up

As shown in Fig. 2, one marker was placed on the spinous process of the first lumbar vertebra (L1), and each innominate was identified by three markers placed on the posterior superior iliac spine (PSIS), the anterior superior iliac spine (ASIS), and the lateral iliac tubercle. A three-armed triangular wand (with arms measuring 1 cm) with a single marker attached to each of the angles was fixed to the second sacral spinous process (S2).

All the markers were directly applied to the skin using double-sided adhesive tape while the participants were in a standing position.

The motion analysis system was calibrated by a designated laboratory expert before each measurement session using a wand with reflective markers and by setting up the reference point within the capture volume. Marker placement was performed by a trained physiotherapist familiar with anatomical landmarks to minimize marker placement and measurement errors.

After preparation and familiarization, the participants stood barefoot standing and performed three forward flexions of the trunk to the end of the possible range and back to the resting position at their preferred speed. Forward flexion of the trunk was chosen for this assessment because it is one of the functional movements that people perform frequently during daily activities and, on the other hand, it produces full range of motion of the sacroiliac joint, which is also used in clinical tests to diagnose SIJ disorders. Data collection was carried out over a period of six month in 2020 conducted in a single session without follow-up.

The SIJ movements were tracked by a 3D motion analysis system (VICON MX; Oxford Metrics, Oxford, England) using 10 video cameras at a sampling frequency of 120 Hz arranged in a laboratory to record the spatial coordinates of each marker attached to the skin overlying the body landmarks.

Data collection was carried out at the gait laboratory of the Djavad Mowafaghian Research Center for Intelligent Neurorehabilitation Technology at Sharif University of Technology.

The 3D motion of each marker during the task was recorded by the Vicon motion analysis system, and the raw data were subsequently imported into MATLAB (MATLAB and Statistics Toolbox Release 2017b, The MathWorks, Inc., Natick, Massachusetts, USA). 3D angles were extracted by a Cardan XYZ (flexion/extension-lateral bend-axial twist) rotation sequence. The Cardan rotation sequence XYZ involves three steps: first, rotation about the laterally directed axis (X (flexion/extension)); second, rotation about the anteriorly directed axis (Y (lateral bend)); and third, rotation about the vertical axis (Z (axial twist)). After LCS (local coordinate system) computation for each segment, the resulting orientation matrix was used for extracting 3D angles. The angles for the XYZ sequence are designated α (alpha) for the first rotation, β (beta) for the second rotation, and ϒ (gamma) for the third rotation. The rotation matrix R and α angle for an XYZ rotation sequence are as follows [31, 32]:

$$\:\text{R}=\left[\begin{array}{ccc}\text{cos}\gamma\:\text{cos}\beta\:&\:\text{cos}\gamma\:\:\text{sin}\beta\:\:\text{sin}\alpha\:+\text{sin}\gamma\:\:\text{cos}\alpha\:&\:\text{sin}\gamma\:\:\text{sin}\alpha\:-\text{cos}\gamma\:\text{sin}\beta\:\:\text{cos}\alpha\:\\\:-\text{sin}\gamma\:\:\text{cos}\beta\:&\:\text{cos}\alpha\:\:\text{cos}\gamma\:-\text{sin}\gamma\:\text{sin}\beta\:\text{sin}\alpha\:&\:\text{sin}\gamma\:\:\text{sin}\beta\:\text{cos}\alpha\:+\text{cos}\gamma\:\text{sin}\alpha\:\\\:\text{sin}\beta\:&\:\text{cos}\beta\:\:\text{sin}\alpha\:&\:\text{cos}\beta\:\:\text{cos}\alpha\:\end{array}\right]$$

$$\:{\alpha\:}={\text{tan}}^{-1}\left(\frac{{-R}_{32}}{{R}_{33}}\right)$$

All motion parameters were extracted at the maximum sagittal displacement of the L1 marker relative to the S2 markers during forward flexion. We quantified the translational and angular differences of the sacrum relative to each innominate (both sides) in the three planes of movement (sagittal, frontal and transverse) before forward flexion of the trunk and the absolute differences between the sacrum translation and rotation relative to the ilium from the initial to final movement stages for subsequent analysis. Intra-observer reliability in three-dimensional kinematic analysis of sacroiliac joint mobility was reported to be strong in the study by Veiga et al. in 2015 [33].

The average of three trials was calculated and used for analysis. The statistical analysis was performed with SPSS version 25.0 (IBM Corp., New York, NY). Kolmogorov-Smirnov test was performed and all variables were distributed normally, so parametric tests were used for analysis. The independent T-test was employed to compare sacral distance related to innominate bone at the standing position and full forward flexion across three planes, as well as to analyze demographic characteristics between groups. Pearson correlation was conducted to assess the relationship between TMD side, severity and pain with sacroiliac joint movements. The characteristics of the subjects are presented as the mean and standard deviation (SD). A 95% confidence interval was used for statistical analysis (*p* < 0.05).

## Results

Forty participants (aged from 20 to 40 years) were included in this study without sample attrition. The characteristics of the subjects are shown in Table 1.

**Table 1:** Characteristics of the subjects (20 healthy and 20 patients with TMD)

Variable	Group	Lowest	Highest	Mean	SD	*P*
**Age** **(year)**	Healthy	20	40	26.55	4.81	**0.31**
	Patient	20	40	28.25	5.76	
**Height** (**cm**)	Healthy	162	186	173.55	6.16	**0.52**
	Patient	162	187	172.05	8.33	
**Weight** (**kg**)	Healthy	52	104	67.7	11.86	**0.86**
	Patient	46	94	68.4	13.14	
**BMI** **(kg/m²)**	Healthy	17.78	30.06	22.39	3.03	**0.56**
	Patient	17.52	29.05	22.98	3.41	
**Sex** **(number)**	Healthy	Male: 12 Female: 8				**0.75**
	Patient	Male: 11 Female: 9				

Intrarater reliability was assessed and found to be good to excellent (ICC between 0.61 and 0.98), and the data was previously published [34]. The results were interpreted as indicating excellent reliability (ICC ≥ 0.75), good reliability (0.40 < ICC < 0.75), and poor reliability (ICC ≤ 0.40) [35].

According to the Fonseca scale for the severity of TMD, we had 8 individuals with mild TMD, 11 with moderate, and 1 with severe TMD in the patients’ group.

Among the TMD group, 7 individuals had right-sided, 9 individuals had left-sided, and 4 individuals had bilateral involvement of ADDR. Pearson correlation showed no relationship between the side of involvement and sacroiliac joint kinematics.

There was no statistical difference between groups for any TMJ movements. *P* values for mouth opening, right and left deviations, and protrusion are as follows respectively: 0.95, 0.11, 0.64, and 0.52. Furthermore, there was no significant association between TMJ range of motion and SIJ kinematics.

Between-group comparison revealed a significant difference in sacral translatory location in the sagittal plane to the innominate bones and rotatory position around the frontal axis on both sides at the starting position before forward flexion of the trunk. No other translatory (on the frontal and vertical plane) or rotatory position (around the sagittal and vertical axis) was found significantly different between the groups, the results are shown in Table 2.

**Table 2:** Kinematics of the SIJ in start position before forward flexion of the trunk

Variable	Relative to	Group	mean	SD	*p*
**Translatory location of sacrum in sagittal plane (mm)**	Right ilium	Healthy	95.93	7.79	**0.001**
		Patient	113.33	12.31	
	Left ilium	Healthy	94.69	3.58	**0.001**
		Patient	108.6	9.85	
**Translatory location of sacrum in frontal plane (mm)**	Right ilium	Healthy	110.79	5.03	0.99
		Patient	110.77	5.64	
	Left ilium	Healthy	98.48	12.07	0.1
		Patient	106.05	7.08	
**Translatory location of sacrum in vertical plane (mm)**	Right ilium	Healthy	47.53	19.56	0.42
		Patient	40.62	17.98	
	Left ilium	Healthy	28.19	11.39	0.44
		Patient	23.75	13.94	
**Rotatory position of sacrum around frontal axis (Y-axis) (°)**	Right ilium	Healthy	67.79	6.43	**0.001**
		Patient	54.95	7.14	
	Left ilium	Healthy	76.89	6.28	**<0.001**
		Patient	56.98	8.67	
**Rotatory position of sacrum around sagittal axis (X-axis) (°)**	Right ilium	Healthy	125.23	11.15	0.06
		Patient	116.17	9.54	
	Left ilium	Healthy	134.51	18.97	0.05
		Patient	120.35	5.33	
**Rotatory position of sacrum around vertical axis (Z-axis) (°)**	Right ilium	Healthy	112.86	2.63	0.79
		Patient	113.28	4.35	
	Left ilium	Healthy	113.01	3.05	0.52
		Patient	113.97	3.64	

No significant difference was found between two groups in sacral translatory or rotatory movements on three planes relative to ilium from starting position to the ending position (forward flexion of the trunk), the results are shown in Table 3.

**Table 3:** Kinematics of the SIJ (changes from standing position to the end of forward flexion of the trunk)

Variable	Relative to	Group	mean	SD	*p*
**Translation of sacrum in sagittal plane (mm)**	Right ilium	Healthy	4.99	3.47	0.3
		Patient	6.18	4.2	
	Left ilium	Healthy	5.44	3.34	0.64
		Patient	5.99	4.78	
**Translation of sacrum in frontal plane (mm)**	Right ilium	Healthy	5.26	2.56	0.49
		Patient	4.63	3.72	
	Left ilium	Healthy	4.19	1.96	0.06
		Patient	3.05	1.44	
**Translation of sacrum in vertical plane (mm)**	Right ilium	Healthy	4	2.08	0.06
		Patient	5.16	3.67	
	Left ilium	Healthy	4.32	2.78	0.34
		Patient	5.03	3.62	
**Rotation of sacrum around frontal axis (Y-axis) (°)**	Right ilium	Healthy	3.09	2.66	0.41
		Patient	1.13	0.51	
	Left ilium	Healthy	1.44	1.14	0.57
		Patient	1.27	0.58	
**Rotation of sacrum around sagittal axis (X-axis) (°)**	Right ilium	Healthy	2.26	1.07	0.44
		Patient	2.02	0.98	
	Left ilium	Healthy	2.47	1.1	0.13
		Patient	1.96	1.03	
**Rotation of sacrum around vertical axis (Z-axis) (°)**	Right ilium	Healthy	4.17	1.74	0.09
		Patient	3.39	0.89	
	Left ilium	Healthy	4.36	1.31	0.05
		Patient	3.37	1.12	

The severity of TMD was associated with translatory location of the sacrum in the sagittal plane and rotatory position of the sacrum around the frontal axis on both sides in starting position before forward flexion of the trunk (*p* < 0.05) as shown in Table 4. However, no significant association was found between TMD severity and other sacral movement at either the starting position or at the end of the task (forward flexion of the trunk).

**Table 4:** Association between TMD severity and SIJ kinematics at the starting position before forward flexion of the trunk in patient’s group

Variable		Relative to	*P* value	*r*
**TMD severity**	**Translatory location of sacrum in sagittal plane (mm)**	Right ilium	**0.006**	**0.52**
		Left ilium	**0.01**	**0.58**
	**Translatory location of sacrum in frontal plane (mm)**	Right ilium	0.72	0.07
		Left ilium	0.29	0.33
	**Translatory location of sacrum in vertical plane (mm)**	Right ilium	0.19	0.36
		Left ilium	0.58	0.38
	**Rotatory position of sacrum around frontal axis (Y-axis) (°)**	Right ilium	**0.003**	**0.79**
		Left ilium	**0.001**	**0.8**
	**Rotatory position of sacrum around sagittal axis (X-axis) (°)**	Right ilium	0.12	0.4
		Left ilium	0.09	0.41
	**Rotatory position of sacrum around vertical axis (Z-axis) (°)**	Right ilium	0.26	0.22
		Left ilium	0.58	0.26

No significant association was found between TMD pain and sacral movements either at the starting position or at the end of the task.

Fisher’s Exact test revealed no significant between-group differences in the presence of hamstring or piriformis muscle tightness (*P* = 1.000).

## Discussion

A comparison of the sacroiliac joint position between the two groups at the two stages (starting position and at the end of the task [forward flexion of the trunk]) showed that there was a significant difference at the starting position in linear (in the sagittal plane) and angular location (around the frontal axis) of the sacrum compared to the ilium on both the right and left sides, but there was no significant difference between groups in other variables either at the starting position or at the end of the task.

Additionally, the results showed that the severity of TMD was associated with linear (in the sagittal plane) and angular location (around the frontal axis) of the sacrum compared to each ilium (right and left) at the starting position, but there was no significant association in other variables either at the starting position or at the end of the task. Additionally, pain in TMJ had no significant association with SIJ kinematics either at the starting position or at the end of the task.

The flexibility of the hamstring and piriformis muscles can impact both the range of motion during forward bending of the trunk and the kinematics of the sacroiliac joint. However, our result did not show any significant differences in these variables between the groups. Therefore, muscle tightness did not appear to be a confounding factor in this study.

At the starting position, the TMD group showed a significantly greater linear distance (translatory location) of the sacrum relative to the ilium on the sagittal plane on both sides (right and left). In contrast, the angular position of the sacrum relative to the frontal axis on both sides (right and left) was significantly greater in healthy group, indicating a greater sacral flexion relative to the ilium on both sides in the healthy group than in the TMD group. These results show that TMDs, especially articular disc disorders (the most common anterior disc displacement with reduction), can be associated with altered kinematics of the sacroiliac joint, meaning that in TMD patients, there are changes in SIJ kinematics that are significantly different from those in healthy individuals; however, it cannot be determined whether TMD was the cause of these changes or the result.

We did not find any significant differences between groups regarding sacral position in other planes (frontal or transverse). This may be attributed to the limited degrees of freedom of the sacrum, which primarily operates in the sagittal plane around the frontal axis [36], as a result, significant differences were not observed in other directions or planes.

These non-significant findings suggest that the sacral movement in the frontal and transverse planes is less influenced by the factors assessed in this study, possibly due to its anatomical constraints. This limitation in movement range may contribute to the absence of significant changes in these planes. Therefore, while the sagittal plane remains the primary axis for sacral motion, future studies could explore other contributing factors that might affect sacral kinematics in the frontal and transverse planes, such as compensatory mechanisms or additional musculoskeletal variables.

Contrary to the starting position, we observed no significant difference between the two groups in any variable acros all planes at the end of the task (forward flexion of the trunk). This finding could suggest that dynamic tasks, such as forward flexion, might minimize initial differences (at the starting position) in sacral alignment between groups, potentially due to biomechanical adjustments or stabilization mechanisms. This result does not align with the findings of Fink et al. [20] who reported hypomobility in the cervical and sacroiliac joints in simulated TMD. Our findings did not reveal a significant difference between the groups, although the severity of TMD was associated with sacral placement in the sagittal plane while standing, more severe TMD corresponded to greater sacral displacement. It is important to note that Fink et al. employed a simulated TMD condition, which may not accurately reflect the effects of actual TMDs. In contrast, the present study examined individuals with chronic TMD. Chronic conditions, along with the body’s compensatory mechanisms, can lead to musculoskeletal adaptations over time [37]. A cross-sectional simulation of TMD, however, does not seem to fully replicate these long-term compensatory adaptations, which may limit its ability to accurately reflect the musculoskeletal changes associated with chronic TMD.

Moreover, they relied on palpation-based clinical tests to assess sacroiliac joint motion, which have raised concerns about their reliability and validity in previous studies [36, 38]. In contrast, the present study used a motion analysis system to assess SIJ motion, which has been shown to offer high accuracy and reliability. These methodological and population differences highlight the need for cautious interpretation when comparing results across studies.

One hypothesis is that the differences observed between groups in the starting position of the task may stem from variations in the initial alignment of the sacrum. During trunk flexion, the sacrum undergoes nutation and stabilizes between the two ilia [39]. This stabilization, coupled with potential biomechanical adjustments and dynamic stabilization mechanisms occurring during movement, likely minimizes the initial differences, which may explain the absence of significant differences at the end of the task.

The Pearson correlation showed that as the TMD severity increased, patients experienced more pain (*p* = 0.001, *r* = 0.68), which was consistent with a previous study indicating that TMD severity was associated with the pressure pain threshold over the masseter muscle [40]. According to the Fonseca scale two of the ten parameters that determine the severity of TMD are directly related to pain in the jaw area and surrounding muscles; therefore, pain intensity and TMD severity have a direct relationship, and the results of the present study confirm this relationship.

Moreover, we found that the severity of TMD was associated with both sacral translatory and angular position in the sagittal plane at the starting position. A greater severity of TMD was associated with greater sacral flexion and less translatory distance in the sagittal plane relative to the ilium. These results were in agreement with the results of Wiest et al. [41] in which they found greater spine curvature in presence of TMD. They investigated the relationship between the severity of TMD and the degree of neck lordosis, thoracic kyphosis, and pelvic tilt and found that these variables increase with increasing TMD severity. Additionally, in the study of Kim et al. [42] a strong relationship between the severity of TMD and the severity of spinal pain was observed. As the group factor did not affect other sacroiliac joint kinematic variables, TMD severity did not significantly affect other variables in the patient group.

Furthermore, no significant association was found between TMD pain and SIJ kinematics in our study. One possible explanation is that TMD pain may not directly affect SIJ motion. Pain intensity did not significantly affect TMJ range of motion in the patient group, nor was there a significant difference in TMJ range of motion between the right and left sides in the TMD group. This finding was consistent with the results of the study by Paula Gomez et al. [43] Since pain intensity did not affect TMJ range of motion, it seems logical that it is not related to the SIJ kinematics, which is a segment more distal to the TMJ.

The temporomandibular joint range of motion was similar between the healthy and patient groups. Since the sample in the patient group in this study had anterior disc displacement with reduction and was classified as no movement limitation type of TMD, it looks reseanable to observe that the ROM of the TMJ was not significantly different between the two groups. Also, the relationship between TMJ range of motion and pelvic kinematics was not significant. Since TMJ range of motion did not differ significantly between the two groups, it is unsurprising that there would be no relationship between TMJ range of motion and pelvic kinematics.

This study acknowledges several limitations. The small sample size of 20 participants per group may affect the generalizability of the findings, and the exclusion of individuals with abnormal spinal curves, while necessary for the study’s focus, may introduce selection bias. Potential errors in marker placement during motion analysis are also recognized; however, examiner training, adherence to standardized marker placement protocols, and repeated trials were implemented to enhance reliability. Finally, the cross-sectional design of the study limits the ability to establish causal relationships, which is an inherent limitation of such study designs.

## Conclusions

In summary, Findings of this study suggest an association between TMD and SIJ kinematics during forward trunk flexion test, which was consistent with some previous studies; Therefore, the results of this study support the need to evaluate the pelvic region specifically in sagittal plane and around the frontal axes as part of the clinical evaluation protocol in TMD patients with anterior disc displacement with reduction (ADDR).

## Data Availability

The datasets generated and analyzed during the current study are available from the corresponding author on reasonable request.

## Abbreviations

TMD: Temporo mandibular disorder
TMJ: Temporo mandibular joint
SIJ: Sacroiliac joint
ADDR: Anterior disk displacement with reduction
DC/TMD: Diagnostic criteria for temporomandibular disorders
BMI: Body mass index
ROM: Range of motion
VAS: Visual analog scale
PSIS: Posterior superior iliac spine
ASIS: Anterior superior iliac spine
SD: Standard deviation
